# Optimal Surgical Approaches for Incidentally Discovered Ruptured Mucinous Appendiceal Neoplasms

**DOI:** 10.7759/cureus.70808

**Published:** 2024-10-04

**Authors:** Mena Louis, Brian Gibson, James Chambers

**Affiliations:** 1 General Surgery, Northeast Georgia Medical Center Gainesville, Gainesville, USA; 2 Trauma and Acute Care Surgery, Northeast Georgia Medical Center Gainesville, Gainesville, USA; 3 General Surgery, Northeast Georgia Medical Center Braselton, Braselton, USA

**Keywords:** appendiceal cancer, hipec, incidental appendiceal mucocele, mucinous neoplasms, ruptured appendix

## Abstract

Mucinous neoplasms of the appendix, including low-grade appendiceal mucinous neoplasms (LAMNs), are rare but significant due to their potential for peritoneal dissemination. These tumors are often discovered incidentally during imaging or surgery for unrelated conditions. The presence of mucinous material in the peritoneum raises concern for pseudomyxoma peritonei, necessitating careful intraoperative and postoperative management.

A 52-year-old male presented with testicular pain, leading to a diagnosis of seminoma. Staging computerized tomography (CT) revealed a dilated appendix with a surrounding fluid collection, suggestive of a ruptured mucocele. Exploratory laparotomy uncovered a large mucinous mass encasing the appendix, with mucinous deposits in the peritoneum. Final pathology confirmed an invasive mucinous adenocarcinoma, well-differentiated, arising in the background of a low-grade mucinous appendiceal neoplasm. The invasive component extended into the subserosa (pT3), while the LAMN component involved the serosa (pT4a).

When mucin is found intraoperatively, surgeons should consider appendectomy with possible conversion to an open approach for thorough exploration. Emergency HIPEC is unnecessary; instead, it should be planned electively after complete cytoreduction. The prognosis depends on the extent of the disease and the success of cytoreductive surgery with hyperthermic intraperitoneal chemotherapy (HIPEC).

## Introduction

Ruptured acute appendicitis is a common surgical emergency, often presenting with a dilated appendix and adjacent fluid collection on imaging [1]. These findings typically prompt urgent surgical intervention to prevent complications such as peritonitis and abscess formation [2]. However, similar imaging and intraoperative appearances can also be seen in cases of a ruptured appendiceal mucocele, a rare but significant condition that arises from mucinous neoplasms of the appendix [3]. The challenge lies in distinguishing between these two entities, as they require different surgical approaches and have distinct prognostic implications [4].

Surgeons must be aware that what initially appears to be a straightforward case of ruptured appendicitis could, in fact, be a ruptured mucocele [5]. The presence of a mucocele, especially if it has ruptured, demands a more cautious and comprehensive approach [6]. Intraoperative findings of mucinous material within the abdominal cavity should raise suspicion for a mucinous neoplasm, and surgeons should be prepared to convert to an open procedure if necessary [7]. The goal is to carefully resect the affected appendix and any associated mucinous deposits to minimize the risk of peritoneal dissemination, which could lead to pseudomyxoma peritonei, a condition with significant long-term morbidity [8].

If the diagnosis of a mucinous neoplasm is made postoperatively on the pathology specimen, prompt multidisciplinary evaluation is crucial [9]. Identifying a ruptured appendiceal mucocele, particularly in cases where mucinous material is found within the peritoneum, necessitates further management, often including cytoreductive surgery and hyperthermic intraperitoneal chemotherapy (HIPEC) [10,11]. Understanding the potential for misdiagnosis and being prepared to adjust the surgical plan accordingly are essential steps in optimizing patient outcomes when dealing with these complex and often insidious conditions [12].

## Case presentation

A 52-year-old male presented to the emergency department with a one-week history of dull, aching right testicular pain and a palpable mass in the right testicle. He reported progressive swelling but denied systemic symptoms. His family history was notable for testicular cancer in a brother, colon cancer in his father, and lung cancer in his grandmother. A scrotal ultrasound revealed multiple masses in the right testicle, prompting a right radical inguinal orchiectomy. Pathology confirmed seminoma with lymphovascular invasion, and staging CT scans were ordered.

**Figure 1:**
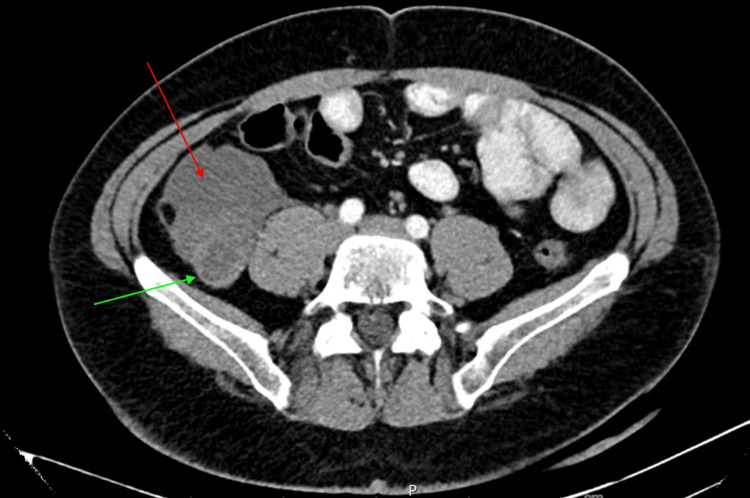
CT abdomen and pelvis with IV and oral contrast, axial image, demonstrates a significantly dilated appendix (marked by a green arrow), measuring up to 2.4 cm in diameter, indicative of an abnormal distension. Adjacent to the appendix, a complex fluid collection (highlighted by the red arrow) is noted, raising concern for a ruptured appendicitis or a ruptured appendiceal mucocele.

**Figure 2:**
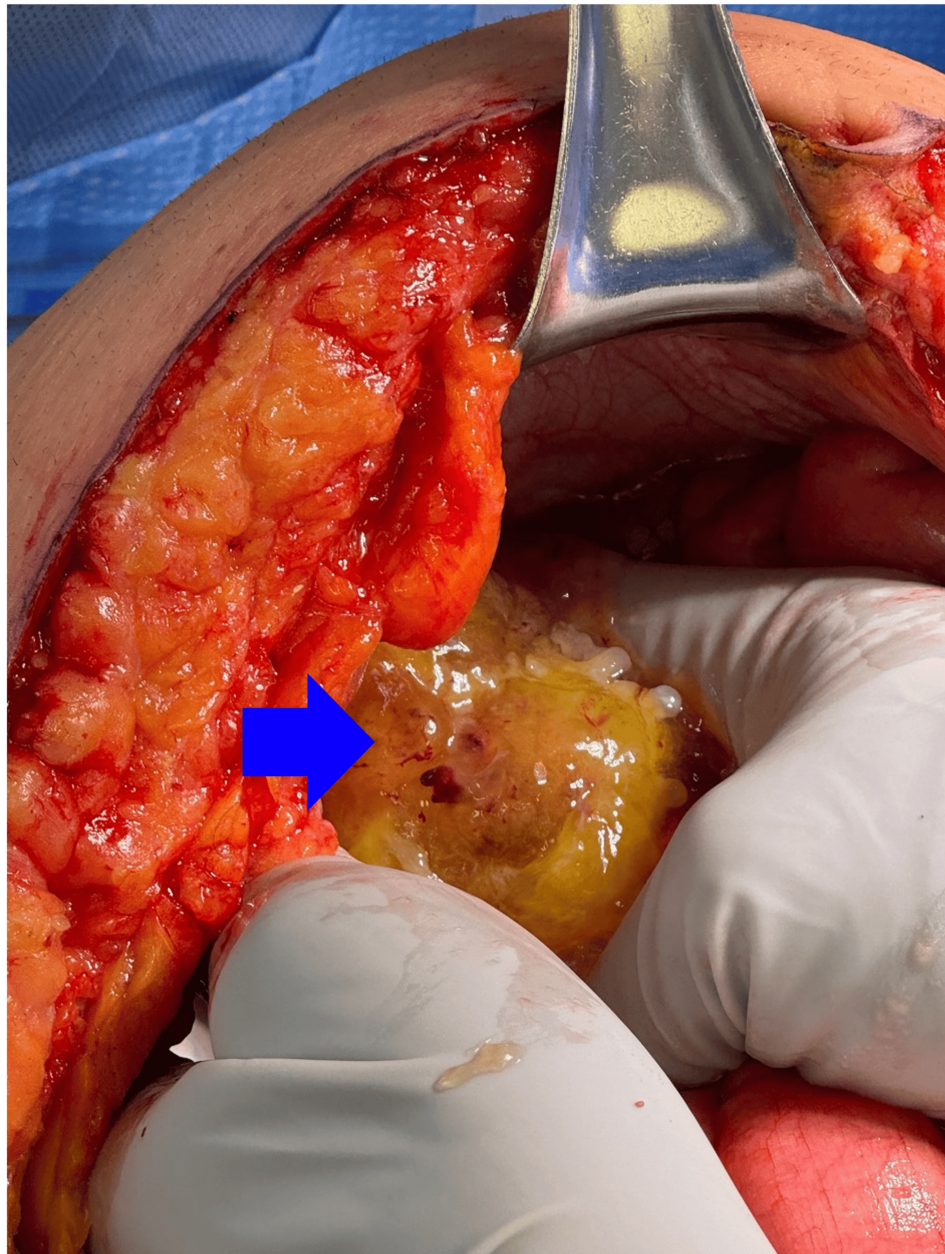
Intraoperative image shows a large mucinous mass in the right lower quadrant, marked by a blue arrow, encountered upon entry into the abdomen.

Two weeks post-orchiectomy, a staging CT of the abdomen incidentally revealed a dilated appendix with disruption of the anterior wall and a surrounding complex fluid collection, suggestive of a ruptured appendicitis or appendiceal mucocele (Figure 1). Despite being asymptomatic, the patient was referred for further evaluation. Exploratory laparotomy revealed a large mucinous mass in the right lower quadrant encasing the appendix and involving the peritoneum (Figure 2). An appendectomy and peritoneal stripping were performed.

Final pathology revealed an invasive mucinous adenocarcinoma arising in the background of a low-grade mucinous appendiceal neoplasm (LAMN). The invasive component extended into the subserosa, classifying it as pT3, while the LAMN component involved the serosa, classifying it as pT4a. The proximal margin was negative for neoplasia. Given these findings, the patient is scheduled to undergo HIPEC to address the residual disease.

## Discussion

Appendiceal mucoceles are rare, typically slow-growing lesions that arise from the appendix, characterized by an accumulation of mucin within the appendiceal lumen [8,13]. These can range from benign retention cysts to malignant mucinous adenocarcinomas [14]. The most significant concern with appendiceal mucoceles is their potential to rupture, leading to the dissemination of mucinous material throughout the peritoneal cavity, which can result in PMP [15]. This condition, if untreated, can lead to extensive abdominal involvement and severe morbidity [16]. The rarity and often nonspecific presentation of appendiceal mucoceles make them challenging to diagnose preoperatively, with many cases being discovered incidentally during surgery for other conditions [17].

When a mucocele is identified intraoperatively, surgeons must carefully evaluate the lesion [18]. If the mucocele appears intact, an appendectomy may suffice, but if there is any indication of rupture or if mucinous material is found within the peritoneal cavity, the surgical approach must be escalated [8]. In such cases, conversion to an open procedure is often recommended to allow for thorough exploration, complete resection of the appendix, and careful removal of any mucinous deposits [19]. The primary goal is to prevent the further spread of mucinous material and to minimize the risk of PMP [4]. Additionally, any suspicious tissue should be sent for frozen section analysis to guide further intraoperative decision-making [20].

Once the appendix and any visible mucinous material have been removed, the surgeon must consider the extent of the disease and the need for further surgical intervention [21]. If the mucinous material appears limited, a thorough washout of the peritoneal cavity may be sufficient [22]. However, if there is extensive mucin spread, an initial debulking may be performed, but the definitive treatment would involve CRS and HIPEC at a later date [23]. HIPEC is not typically performed during the initial surgery unless the patient is already in a specialized center with the necessary resources and personnel available [24]. Instead, patients should be stabilized, and a multidisciplinary team should be consulted postoperatively to plan for CRS and HIPEC [12].

The prognosis for patients with appendiceal mucoceles, particularly those with malignant transformation or extensive peritoneal involvement, varies significantly based on the stage at diagnosis and the success of initial treatment [25]. Patients who undergo complete cytoreduction and HIPEC generally have a more favorable prognosis, with a lower risk of recurrence and longer survival [26]. However, those with incomplete cytoreduction or advanced disease at presentation face a higher risk of recurrence and poorer outcomes [27]. Long-term follow-up is essential for these patients, with regular imaging and clinical assessments to monitor for recurrence [9,27]. The multidisciplinary team should also consider the potential need for additional surgeries or repeat HIPEC if recurrence occurs [28]. Early detection and comprehensive management are key to improving outcomes for patients with this challenging condition.

## Conclusions

The intraoperative discovery of a ruptured appendiceal mucocele during appendectomy or exploratory laparotomy necessitates immediate adjustment to the surgical approach to prevent the spread of mucinous material and subsequent development of PMP. Surgeons should convert to an open procedure if necessary, carefully resect the appendix, and remove all visible mucinous deposits. Postoperatively, a thorough follow-up plan involving regular imaging is crucial. Patients should be referred to specialized centers for consideration of CRS and HIPEC to improve long-term outcomes. Early and aggressive management is key to reducing recurrence and enhancing survival in these patients.
